# Arthroscopic treatment of a lipoma arborescens of the elbow: A case report

**DOI:** 10.1097/MD.0000000000023595

**Published:** 2020-12-11

**Authors:** Joris Paccaud, Gregory Cunningham

**Affiliations:** a Department of Orthopaedics and Trauma Surgery, Geneva University Hospitals, Switzerland, Rue Gabrielle-Perret-Gentil 4; b Shoulder Center, Hirslanden Clinique la Colline, Geneva, Switzerland.

**Keywords:** arthroscopic treatment, elbow, elbow arthroscopy, humeroulnar impingement, lipoma arborescens

## Abstract

**Introduction:**

Lipoma Arborescens is a rare pathology that mainly affects the knee. Occurrences in the elbow are even more uncommon and mainly involve the bicipitoradial bursa.

**Case's description:**

We describe the case of a 54-year-old patient known for rheumatoid arthritis, who consulted for chronic elbow pain associated with swelling and limited extension.

**Diagnosis:**

The diagnosis of a lipoma arborescens of the elbow involving the whole joint was made using magnetic resonance imaging and confirmed during arthroscopy.

**Interventions:**

After a failed nonoperative treatment consisting in intra-articular cortisone injections and physiotherapy, the patient underwent arthroscopic synovectomy and arthrolysis.

**Outcome:**

At 1-year follow-up, he reported no pain, satisfactory range of motion, and major improvements in clinical scores.

**Conclusion:**

This is the first illustrated case report about lipoma arborescens involving the whole elbow joint. Even though it is a rare disease, awareness of its presentation, imaging patterns, and treatment options is therefore important for clinicians, radiologists, and surgeons. In this case, arthroscopic treatment resulted in satisfactory and long-lasting pain relief and functional results. It may be considered as a safe and effective option in case of failed nonoperative measures.

## 1 Introduction

Lipoma arborescens (LA) is a rare pathology, consisting in adipose metaplasia of the subsynovial tissue arising from chronic joint inflammation. It has mainly been reported to occur in the suprapatellar bursa but may also involve other joints, such as the shoulder, ankle, or elbow.^[1,2]^ In the latter, the few reports in the current literature mainly involve the bicipitoradial bursa and only one case report documents the outcome after open surgery for such manifestation.^[3]^ We present an unusual case of a 54-year-old man with a history of rheumatoid arthritis (RA) presenting with a severe refractory LA involving the whole elbow, causing mechanical posterior humeroulnar impingement, successfully treated with arthroscopic synovectomy and olecranon plasty. This is to the best of the authors’ knowledge the first fully reported and illustrated case about arthroscopic treatment of elbow LA.

### 1.1 Patient consent statement

The patient has given his informed consent regarding the publication of this case.

## 2 Case presentation

**Figure 1:**
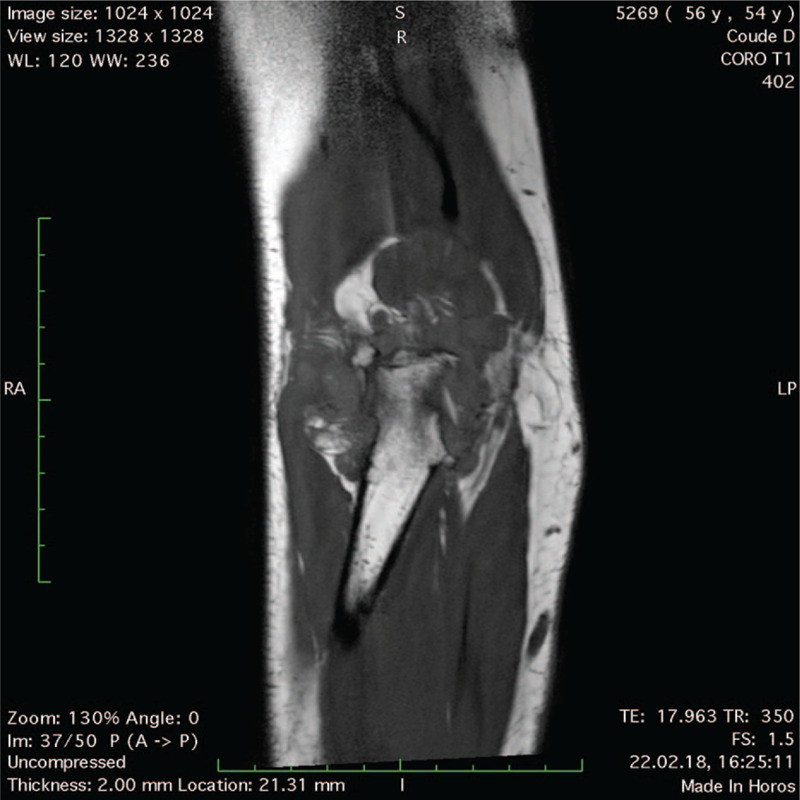
MRI T1 sequence coronal view of the right elbow showing intra-articular LA involving the whole joint.

**Figure 2:**
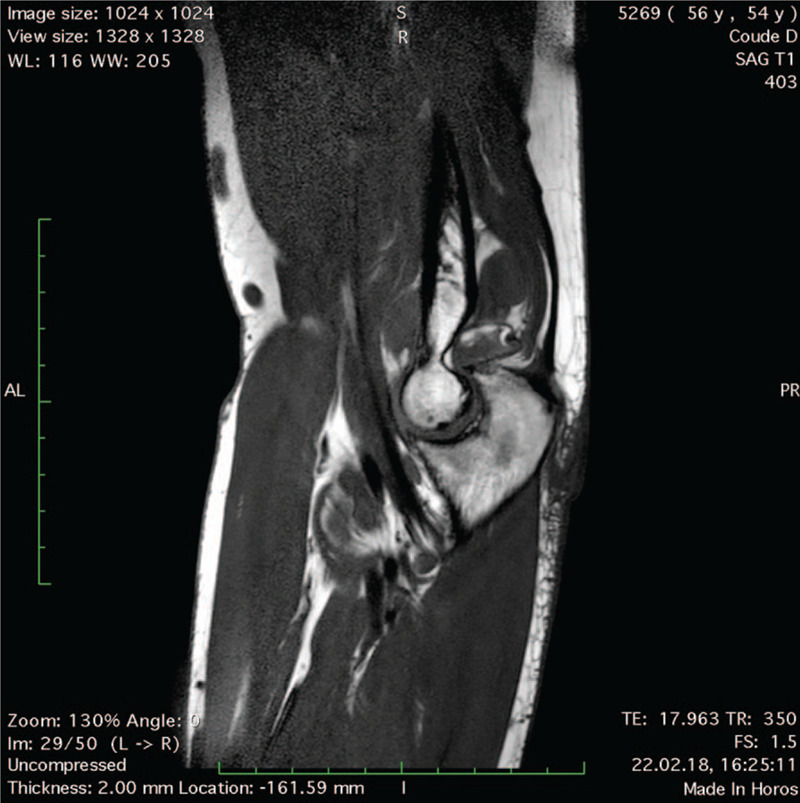
MRI T1 sequence sagittal view of the right elbow showing posterior humeroulnar impingement caused by LA.

**Figure 3:**
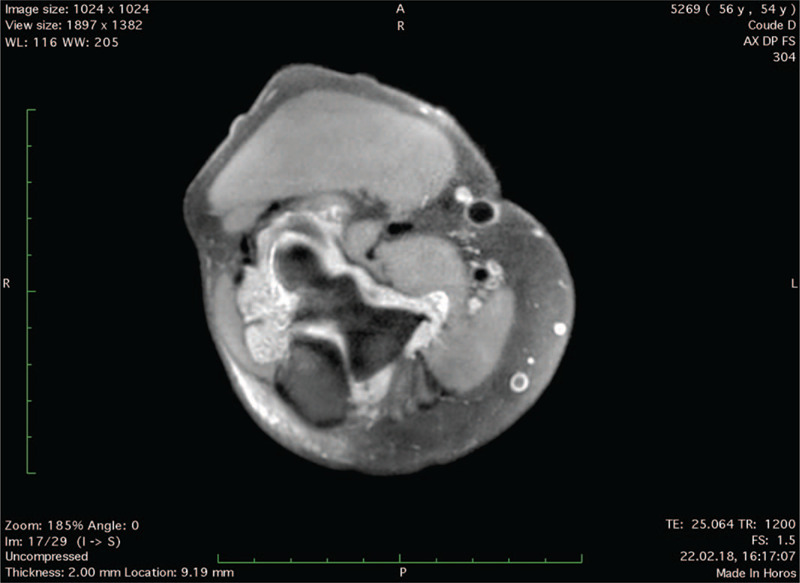
MRI DP FS sequence axial view of the right elbow showing intra-articular LA villi.

A 54-year-old right-handed male patient known for RA treated with Methotrexate and anti-TNF-α was referred to a specialized shoulder and elbow clinic for right chronic elbow pain refractory to conservative management, consisting in intra-articular cortisone injection and physical therapy. He complained about posterior joint pain, swelling, and a deficit in extension, causing severe disability in his daily life and professional activities as a firefighter. Pain Visual Analogic Scale (pVAS) was 8/10,^[4]^ elbow Single Assessment Numeric Evaluation (SANE) score 25/100,^[5]^ Mayo Elbow Performance Score (MEPS) 35/100.^[6]^ Physical examination showed joint effusion with tenderness on palpation of the olecranon fossa, painful restricted range of motion (ROM) with 140–20–0° in flexion-extension compared to 150–0–0° on the contralateral side, pronosupination was unrestricted. There were no signs of ulnar nerve entrapment. Preoperative magnetic resonance imaging (MRI) showed a large intra-articular multilobulated pseudo-tumoral mass (Fig. 1) causing posterior humeroulnar impingement (Fig. 2), with mixed components including lipomatous and synovial fringes (Fig. 3), characteristic of LA. Due to the severity and duration of his disease with failed nonoperative measures, the patient underwent arthroscopic synovectomy and posterior humeroulnar decompression.

## 3 Surgical treatment (see Supplemental File 1 (video of arthroscopic treatment of LA: synovectomy and arthrolysis, 2 min 30 sec, 71 MB))

The patient was operated under general anesthesia in the lateral decubitus with the upper limb draped freely and placed on an arm hold. A humeral tourniquet was inflated at 280 mmHg for 55 minutes. The joint was first distended by injecting 20 ml of saline solution through the lateral soft spot to move away the anterior neurovascular structures. The anterior compartment was inspected through proximal anteromedial and anterolateral portals using a 2.9 mm 30° angulated scope. Tissue samples were taken sent for anatomopathological analysis. LA was predominantly found in the supracapitellar, medial and lateral recesses, as well as in the bicipito-radial space. To access the latter, partial section of the annular ligament was performed in order to drive the scope under it. The hypertrophic adipose villi and inflammatory synovium were resected using a 3.2 mm Shaver until the pale underlying capsuloligamentous structures were fully exposed and carefully preserved. The posterior compartment was then explored using a posterolateral viewing portal and a direct posterior transtricipital working portal. Soft tissues were debrided until the olecranon fossa and olecranon tip were properly exposed. Elbow extension demonstrated an important humeroulnar impingement caused by LA tissue incarcerating in the joint. A thorough debridement and osteophyte resection of the tip of the olecranon using a high-speed 4 mm Burr, allowed gaining full and impingement-free extension. The arthroscopic portals were closed with non-absorbable stitches and the arm immobilized in a temporary sling.

## 4 Postoperative care

The patient was discharged from the hospital on day one and allowed to move his elbow freely as tolerated by pain, with physical therapy twice a week. Dressings were changed and stiches were removed on day 14. Histological analysis confirmed the diagnosis of LA.

He was clinically reassessed after 1.5, 3, 6, and 14 months.

## 5 Outcome

The patient already reported immediate significant improvement in pain range of motion and did not require any postoperative opioids. He was able to go back to his full recreational and professional activities on the 6th postoperative day. At 6 weeks postoperative, pVAS score was 0/10, SANE was 90/100 and MEPS was 85/100. Physical examination showed no more pain on palpation, with a flexion-extension of 150°–20°–0.

At 6 months postoperative, ROM was near complete with a flexion-extension of 150°–5°–0°, pVAS remained at 0/10, SANE at 90/100 and MEPS improved to 100/100.

At latest follow-up after 14 months, the exam and clinical scores remained unchanged. The patient reported high satisfaction and no limitations in his daily life and work activities.

## 6 Discussion

Current literature about LA is scarce and mainly consists in case reports. Interestingly, many of them have been published in the last years^[7,8]^ although this entity was described over 50 years ago.^[9]^ This may be due to an increased awareness about this disease and improvement in modern imaging techniques (MRI). The exact pathophysiology of LA remains poorly understood. It seems to be related to repetitive micro-trauma, systemic conditions such as Diabetes Mellitus, psoriatic arthritis, or RA, as in the presented case.^[10]^ LA is mainly monoarticular, although some cases have reported bilateral joint involvement.^[11]^ It has mostly been reported to involve the suprapatellar recess in the knee, and much more rarely the bicipito-radial bursa in the elbow.^[12]^ This is to the best of the authors knowledge the only report about full elbow joint involvement in LA treated with arthroscopic debridement. Presentation is usually painless but can be associated with recurrent joint swelling and intermittent pain. In this case the entrapment of hypertrophic synovial villi caused symptomatic posterior humeroulnar impingement.

Definitive treatment of symptomatic LA is usually surgical, as it is known to be refractory to nonoperative measures. There is only one reported case about a successful conservative treatment with yttrium-90 radiosynovectomy,^[13]^ but no other reports using this method. According to a systematic review about the treatment of LA in the knee, gold standard treatment is arthroscopic synovectomy with a success rate exceeding 95%.^[14]^ Evidences for LA treatment of the elbow joint are insufficient but it is safe to assume that an arthroscopic approach is also the most effective.

Elbow arthroscopy offers many advantages. Firstly, it allows a meticulous and complete examination of the whole joint, concomitant synovectomy, removal of loose bodies and precise bony resection. It avoids extensive open approaches required to expose the different elbow compartments and preserves the integrity of the surrounding soft tissues, preventing important scarring and potential joint contracture. However, care should be taken as the neurovascular structures are at risk due to their proximity to the joint capsule, especially in severely deformed arthritic elbows. Therefore, the authors advise to preserve the integrity of the underlying capsule in order to avoid iatrogenic neurovascular damage and prevent excessive fluid extravasation which compromises visibility.

Outcome after surgery is generally satisfactory and recurrence is uncommon,^[15]^ although the latter remains formally unknown. In the presented case, the patient showed rapid pain relief and full functional recovery that remained satisfactory after 14 months.

## 7 Conclusion

This is the first illustrated case report about lipoma arborescens involving the whole elbow joint. Even though it is a rare disease, awareness of its presentation, imaging patterns, and treatment options is therefore important for clinicians, radiologists, and surgeons. In this case, arthroscopic treatment resulted in satisfactory and long-lasting pain relief and functional results. It may be considered as a safe and effective option in case of failed nonoperative measures

## Author contributions

Joris Paccaud wrote the manuscript and edited the supplementary data, Gregory Cunningham collected data and wrote the manuscript.

## Supplementary Material

Supplemental Digital Content
